# Unveiling the Role of Human PER3 Gene Polymorphism (rs57875989) as a Potential Risk Factor in Fibromyalgia Syndrome Patients

**DOI:** 10.7759/cureus.75210

**Published:** 2024-12-06

**Authors:** Sidrah Parvez, Andrej Dzupina, Ghizal Fatima, Jan Fedacko, Aminat Magomedova, Abbas A Mehdi

**Affiliations:** 1 Department of Biotechnology, Era University, Era's Lucknow Medical College and Hospital, Lucknow, IND; 2 Department of Cardiology and Angiology, National Institute of Cardiovascular Diseases, Bratislava, SVK; 3 Department of Biotechnology, Era University, Era's Lucknow Medical College and Hospital, lucknow, IND; 4 Department of Cardiology, Pavol Jozef Šafárik University, Kosice, SVK; 5 Department of Population, Lomonosov Moscow State University, Moscow, RUS; 6 Department of Biochemistry, Era University, Era's Lucknow Medical College and Hospital, Lucknow, IND

**Keywords:** clock, fms, pcr, per3, vntr

## Abstract

Purpose

Fibromyalgia syndrome (FMS) presents a chronic pain condition affecting muscles and joints. Investigating circadian rhythms' disruption, integral to physiological responses, this study delves into the potential impact of *PER3 CLOCK* gene polymorphism (rs57875989) on FMS pathogenesis.

Methods

In this study, we investigated *PER3* gene polymorphism in 100 FMS patients and an equal number of control individuals. The genotyping of the *PER3* gene polymorphism was conducted using polymerase chain reaction (PCR) methodology. Subsequently, we evaluated the association between *PER3* gene polymorphism and FMS susceptibility using odds ratios (ORs) and 95% confidence intervals (CIs) by comparing the genotype and allele frequencies of the* PER3* gene polymorphism between FMS patients and controls.

Results

The* PER3* gene revealed three genotypes: 4/4, 4/5, and 5/5, with allele frequencies showing significant associations between FMS patients and controls (p<0.05). Notably, *PER3* gene polymorphism was linked to FMS development, particularly the 4/4 genotype versus the combined 4/5 and 5/5 genotypes (OR=2.85; 95% CI, 1.35-6.0; p=0.008). These findings suggest a potential role of *PER3 *gene variation as a genetic risk factor for FMS.

Conclusion

These findings reported a potential association between *PER3* gene polymorphism and FMS, illuminating novel pathways for comprehending and addressing this complex condition. This study holds promise for advancing our understanding of FMS etiology and may inform the development of innovative management strategies tailored to individual genetic profiles, potentially leading to more effective treatments and improved outcomes for patients grappling with FMS.

## Introduction

Fibromyalgia syndrome (FMS) manifests as widespread musculoskeletal pain, accompanied by stiffness, fatigue, and disrupted sleep patterns [1,2]. The causes and mechanisms underlying this disorder are currently not comprehended, but it is hypothesized that both genetic and environmental variables may contribute to its development [3-5]. Several investigations have found that genetic differences in *CLOCK* genes, which control circadian rhythm, were linked to diurnal preference, sleep-wake disturbances, and mood changes [6]. Disrupted circadian patterns are associated with anxiety and sleep difficulties [7]. FMS patients commonly experience difficulties in sleep, anxiety, and depression. It is hypothesized that variations in the *CLOCK* gene may indirectly influence the clinical characteristics of FMS. This suggests that the genes responsible for controlling the internal clock of the body may have a role in the development of FMS [7].

*PER3*, a crucial component of the circadian clock, plays a pivotal role in regulating biological rhythms and coordinating physiological processes with the external environment. This gene belongs to the protein-binding family and contains Per-ARNT-Sim (PAS) domains essential for the formation of protein dimers. By forming complexes with other circadian proteins such as cryptochrome and period (PER), *PER3* participates in translational-transcriptional feedback loops, exerting a negative effect on circadian gene activation mediated by heterodimeric transcription factors like *CLOCK/BMAL1 *[8]. Moreover, the *PER3* gene harbors a variable-number-tandem-repeat (VNTR) region, which consists of four or five repeating sequences of fifty-four base pairs encoding eighteen amino acids [9]. Several studies have highlighted the significance of *PER3* gene variations in regulating various aspects of human physiology and behavior. For instance, research has demonstrated correlations between *PER3* gene polymorphism and diurnal preferences, sleep/wake homeostasis, and cognitive abilities [10].

Single nucleotide polymorphisms (SNPs) in the *PER3* gene have also been associated with aberrant circadian parameters, chronotypes, and mood disorders such as depression [11,12,13]. Despite these insights into the multifaceted roles of *PER3* gene variations, its specific involvement in the pathogenesis of FMS remains insufficiently understood. FMS is a complex disorder characterized by chronic widespread musculoskeletal pain, fatigue, sleep disturbances, and cognitive impairment. While the etiology of FMS is multifactorial and poorly understood, growing evidence suggests a potential role of genetic factors in its development and progression. To address this knowledge gap, our study aimed to investigate the relationship between FMS and *PER3* gene variations among North Indian women. We focused on North Indian women due to their genetic diversity, reported a higher prevalence of FMS, and the feasibility of recruiting participants from this specific demographic region. We conducted genotyping of *PER3* gene polymorphism using polymerase chain reaction (PCR) methodology in a cohort comprising 100 FMS patients and an equal number of control individuals.

The association between *PER3* gene polymorphism and FMS susceptibility was assessed using statistical measures such as odds ratios (ORs) and 95% confidence intervals (CIs). Our findings revealed significant associations between *PER3* gene polymorphism and FMS, with distinct genotype and allele frequencies observed between FMS patients and controls (p<0.05). Notably, the 4/4 genotype of the *PER3* gene was particularly linked to FMS development compared to the combined 4/5 and 5/5 genotypes, with an odds ratio (OR) of 2.85 and a 95% confidence interval (CI) of 1.35-6.0 (p=0.008). These results suggest a potential genetic predisposition conferred by *PER3* gene variation in the development of FMS among North Indian women. Our study provides novel insights into the role of *PER3* gene polymorphism in FMS susceptibility, highlighting the complex interplay between genetic factors and the pathogenesis of this debilitating syndrome. Further research is warranted to elucidate the underlying mechanisms and potential therapeutic implications of *PER3* gene variation in FMS.

## Materials and methods

This study includes 100 patients diagnosed with FMS and 100 control volunteers from Era's Lucknow Medical College and Hospital (ELMCH), Era University, Lucknow, India. This study has been approved by the Institutional Ethics Committee of the ELMCH. The patients who attended the Rheumatology Department at ELMCH, Lucknow, were included in the study. The study included those FMS patients who fulfilled the specific criteria outlined by the American College of Rheumatology in 2016 [14]. The study excluded participants with rheumatic arthritis, diabetes, systemic lupus, multiple sclerosis, and any other endocrine condition. The control group was volunteers from ELMCH, Lucknow. The control group has no previous record of rheumatic disease and is not taking any drugs at the date of enrollment. FMS patients and healthy controls filled out the questionnaires that inquired about age, weight, height, the ‘Beck depression inventory’ (BDI), the ‘Beck anxiety inventory’ (BAI), sleep-wake disorder, fatigue, headache, stiffness, paresthesia, family history, difficulty in concentrating, present pain on the ‘visual analogue scale’ (VAS), and the ‘fibromyalgia impact questionnaire revised’ (FIQR) (Appendix 1). After taking the written informed consent, the blood samples were obtained from the study participants in the ethylene-diamine-tetra-acetic acid (EDTA) vials and frozen at -40^o^C for up to six months, ensuring sample integrity for subsequent genetic analysis within the study's timeframe. Our study employed matching criteria to ensure comparability between FMS patients and controls, including age (±5 years), gender, and the absence of chronic conditions. For quality control of PCR, negative controls (no template DNA) were included in each run to check for contamination, while amplification efficiency was confirmed using standard curves. Reagents were stored as per manufacturer guidelines, and repeatability was tested with a subset of samples. The questionnaire used for clinical assessment underwent validation through pilot testing in 20 individuals, with reliability measured by a Cronbach’s alpha of 0.85, ensuring internal consistency. Statistical adjustments for multiple comparisons were applied using the Bonferroni correction, setting a significance threshold of p<0.05 for the comparisons made. These measures ensured robust data collection, analysis, and reproducibility of the findings.

Genotyping

The salting out procedure was used to isolate DNA from the blood samples. The *PER3* gene polymorphism was genotyped using the polymerase chain reaction (PCR) approach. The primers were designed using Primer3 software and synthesized by Integrated DNA Technologies (IDT). The forward primer was 5’-TGT CTT TTC ATG TGC CCT TAC TT-3’, and the reverse primer was 5’-TGT CTG GCA TTG GAG TTT GA-3’.The PCR procedure was conducted using a final quantity of 25 µl, which included 150 to 200 ng of the genomic DNA, using 10 pmol each of the primer and 2x master mixture (Takara, Kasatsu, Japan) in each tube. The PCR was carried out using the gradient-based Master-Cycler (Bio-rad, Hercules, California). The PCR protocol consists of an initial denaturation step at 94^o^C for five minutes, followed by 30 cycles of denaturation at 94^o^C for 40 seconds, annealing at 60^o^C for 40 seconds, and extension at 72^o^C for 40 seconds with a final extension at 72^o^C for five minutes. The amplification products were examined using 2.5 percent of agarose-gel electrophoresis. The PCR results showed three distinct genotypes according to the DNA size: *PER3* 4/4 genotype - 347bp, *PER3* 5/5 genotype - 401bp, and *PER3* 4/5 genotype- 347/401bp.

Statistical analysis

The data were analyzed through post hoc analysis using IBM SPSS Statistics for Windows, Version 28 (Released 2021; IBM Corp., Armonk, New York, United States).. The statistical significance of the variances between FMS patients and healthy subjects was assessed using the logistic regression analysis. Furthermore, we evaluated the odds ratio (OR) and the confidence interval for 95% (CI). The study analyzed the differences in genotypes and alleles distribution of the *PER3* gene polymorphism among the study participants using the chi-square test. The Fisher's exact test was applied when necessary. The analyses were conducted using a two-tailed approach, and differences were considered statistically significant if the p-value was less than 0.05.

## Results

**Figure 1:**
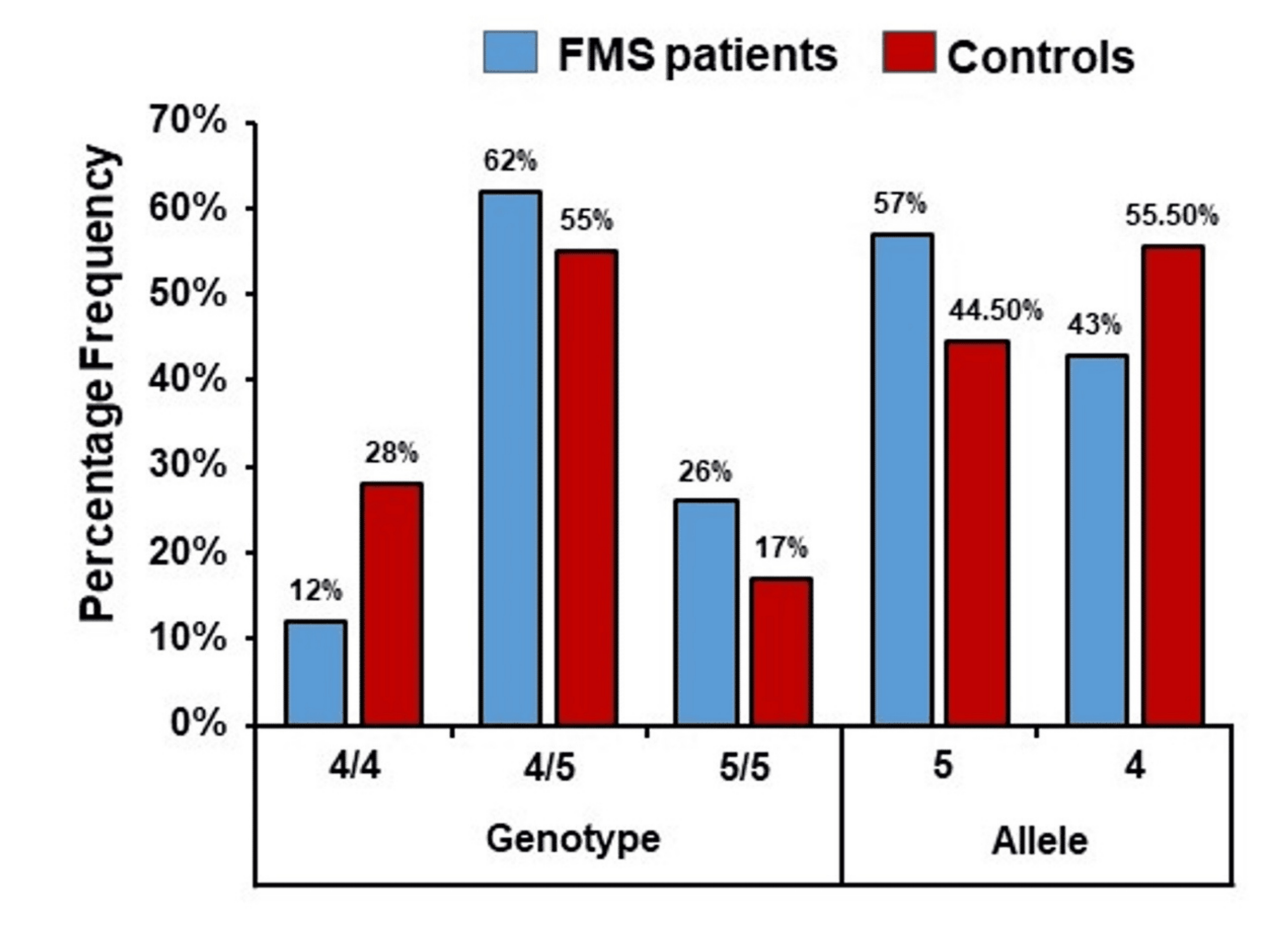
The genotype and allele frequency of PER3 gene polymorphism in the study subjects FMS: fibromyalgia syndrome

The distribution frequency of genotype and allele of the *PER3* gene polymorphism in FMS patients and control groups is mentioned in Table 1. Figure 1 represents a significant difference among the genotype and allele frequencies of the *PER3* gene polymorphism between FMS patients and healthy control subjects. A statistically significant difference was observed among FMS patients and the healthy controls according to the 4/4 vs. 4/5 + 5/5 genotype (OR=2.85; 95% CI, 1.35-6.0; p=0.008). In addition, we conducted an analysis to determine if there were any variations in the clinico-pathological characteristics of FMS patients according to the genotype variations of the *PER3* gene polymorphism (Table 2). There were significant associations between the *PER3* gene polymorphism and clinical characteristics such as sleep-wake disorder and fatigue. *PER3* genotype was associated with FIQR and VAS (p<0.05) (Table 3).

**Table 1:** Genotypic and allelic distributions of PER3 gene polymorphism in FMS cases and controls FMS: fibromyalgia syndrome; OR: odds ratio; CI: confidence interval; p<0.05 is considered significant

Genotype	FMS patients N=100 (%)	Controls N=100 (%)	P-value	OR (95% CI)
4/4	12 (12%)	28 (28%)	0.01	-
4/5	62 (62%)	55 (55%)		
5/5	26 (26%)	17 (17%)		
4/4+4/5:5/5	74:26	83:17	0.16	1.71(0.86-3.41)
4/4:4/5+5/5	12:88	28:72	0.008	2.85(1.35-6.0)
Allele				
5	114 (57%)	89 (44.50%)	0.01	1.65(1.11-2.45)
4	86 (43%)	111 (55.50%)		

**Table 2:** Comparison of PER3 genotype distributions in FMS patients according to accompanying symptoms FMS: fibromyalgia syndrome; SD: standard deviation; BDI: Beck depression inventory; BAI: Beck anxiety inventory; p<0.05 is considered significant

Characteristics	4/4(N=12)	4/5(N=62)	5/5(N=26)	P-value
Age, Mean±SD	35.2±9.9	36.4±9.8	33.7±9.2	0.59
Weight, Mean±SD	60.2±9.0	59.9±9.0	59.0±9.3	0.90
Height, Mean±SD	154.2±4.0	153.6±5.0	153.9±4.6	0.86
BDI, Mean±SD	22.2±12.1	19.6±7.8	19.7±12.6	0.86
BAI, Mean±SD	16±3.5	17.1±1.4	15.1±2.6	0.39
Sleep-wake disorder, n (%)	7(5)	45(16)	10(17)	0.01
Fatigue, n (%)	6(16)	40(22)	9(17)	0.03
Headache, n (%)	1(11)	42(20)	16(10)	0.16
Stiffness, n (%)	7(5)	47(15)	13(13)	0.05
Paresthesia, n (%)	2(10)	14(48)	7(19)	0.77
Family history, n (%)	9(3)	44(18)	14(12)	0.24
Difficulty in concentrating, n (%)	10(2)	41(21)	12(14)	0.06

**Table 3:** Comparison of FIQR and VAS pain score levels and genotype distribution, clinical parameters, and allele frequencies of the PER3 gene in FMS patients and controls FMS: fibromyalgia syndrome; FIQR: fibromyalgia impact questionnaire revised; VAS: visual analogue scale; SD: standard deviation; p<0.05 is considered significant The *PER3* gene polymorphism was analyzed across three genotypes: 4/4, 4/5, and 5/5, with significant associations observed between genotype distributions and fibromyalgia syndrome (FMS). The genotype distribution in FMS patients was 4/4: 12%, 4/5: 62%, and 5/5: 26%, compared to controls (4/4: 40%, 4/5: 45%, and 5/5: 15%, p < 0.05). Allele frequencies: 4 allele: FMS patients: 86% (86/100); Controls: 65% (65/100) 5 allele: FMS patients: 14% (14/100); Controls: 35% (35/100) Significant differences in clinical parameters were also observed. FIQR scores were highest in the 5/5 genotype (92.8 ± 4.2) and lowest in the 4/5 genotype (87.5 ± 12.1, p=0.03). VAS pain scores were highest in the 5/5 genotype (4.4 ± 1.1) and lowest in the 4/5 genotype (3.9 ± 0.5, p=0.01). The 4/4 genotype was significantly associated with increased FMS risk compared to combined 4/5 + 5/5 genotypes (OR=2.85; 95% CI, 1.35–6.0; p=0.008).

Genotype	FIQR (Mean ± SD)	p-value (FIQR)	VAS Pain (Mean ± SD)	p-value (VAS Pain)	Frequency in FMS Patients (%)	Frequency in Controls (%)
4/4	92.2 ± 4.8	0.03	4.3 ± 1.1	0.01	12%	40%
4/5	87.5 ± 12.1		3.9 ± 0.5		62%	45%
5/5	92.8 ± 4.2		4.4 ± 1.1		26%	15%

Allele	Frequency in FMS Patients (%)	Frequency in Controls (%)
4 allele	86%	65%
5 allele	14%	35%

## Discussion

This article investigates the potential link between *PER3* gene polymorphism and the susceptibility to FMS while also assessing its correlation with clinic-pathological outcomes. Specifically, the study identifies that the presence of the five-repeated allele of the *PER3* gene is associated with certain phenotypic traits. These traits include morning-type circadian preferences, cognitive decline following sleep deprivation, alterations in the timing or quantity of cortisol or melatonin secretion, and a propensity towards experiencing depressive symptoms. By elucidating these associations, the research sheds light on the intricate interplay between *PER3* gene variation and clinical manifestations, offering valuable insights into the underlying mechanisms contributing to FMS susceptibility and phenotype variability [15]. This study represents the first investigation into *PER3* gene variations among North Indian FMS patients. Our findings reveal a significant disparity in the distribution frequency of genotypes and alleles of the *PER3* gene between FMS patients and the control group, highlighting its potential relevance in FMS susceptibility. Moreover, we established a correlation between *PER3* gene polymorphism and clinical symptoms, such as sleep-wake disturbances and fatigue in FMS patients. Additionally, the *PER3* VNTR genotype exhibited associations with both the fibromyalgia impact questionnaire-revised (FIQR) and visual analog scale (VAS) scores, indicating its potential role in influencing disease severity and symptomatology.

 Our results align with previous research by Lázár AS et al., which demonstrated a correlation between genetic variations in *PER3* and sleep patterns and preferences for daytime or nighttime activities. Specifically, the *PER3* 5/5 genotype was associated with morning-evening types, while a higher frequency of the *PER3* 4/4 genotype was observed in delayed sleep/wake phase disorder, emphasizing the relevance of *PER3* gene polymorphism in circadian rhythm regulation and its impact on sleep-related disorders [16]. The *PER3* gene regulates circadian rhythms, influencing sleep-wake cycles, hormonal secretion, and cellular processes. Polymorphisms in *PER3* may disrupt its function, leading to desynchronized circadian rhythms. This misalignment can impair sleep quality, reducing restorative sleep, and altering pain perception through changes in melatonin and cortisol levels, which are vital for stress regulation and inflammation control. These disturbances exacerbate fatigue and amplify fibromyalgia symptoms, as insufficient or disrupted sleep heightens central sensitization, a hallmark of FMS. Regarding sleep phenotypes, the *PER3* 5/5 genotype is related to the earlier sleep-wake times [16].

Cheng et al. highlighted a correlation between disrupted diurnal sleep patterns in individuals with the *PER3* 4/4 genotype and heightened vulnerability to stress-induced sleep disturbances. Conversely, they suggested a potential association between disrupted sleep in individuals with the *PER3* 5/5 genotype and the misalignment of their circadian rhythm. These findings underscore the intricate interplay between *PER3* gene polymorphism, sleep patterns, and circadian rhythm regulation, shedding light on potential mechanisms underlying sleep disturbances and their susceptibility to stress-induced disruptions [17]. Viena TD et al. identified that individuals with the *PER3* 4/4 genotype exhibited greater susceptibility to psychological outcomes, particularly state and mood anxiety when compared to individuals carrying longer alleles of the *PER3* gene. This suggests a potential association between *PER3* gene polymorphism and vulnerability to psychological distress, highlighting the role of genetic factors in shaping mental health outcomes [18].

The findings emphasize the importance of considering genetic variations such as the *PER3* genotype in understanding individual differences in susceptibility to anxiety-related disorders. By elucidating the impact of *PER3* gene polymorphism on psychological outcomes, this research contributes to a deeper understanding of the genetic underpinnings of anxiety disorders and may inform personalized approaches to mental health care. Further investigations into the mechanisms underlying the observed associations are warranted to elucidate the complex interplay between genetic factors, psychological states, and susceptibility to anxiety disorders [18]. Nevertheless, our study did not identify any association between *PER3* gene polymorphism and psychiatric assessments, including the Beck depression inventory (BDI) and Beck anxiety inventory (BAI). Despite the observed correlations with other clinical symptoms and outcomes, such as sleep disturbances and fatigue, our findings suggest that *PER3* gene variations may not directly influence psychological distress as measured by these specific psychiatric tests. This underscores the complexity of the relationship between genetic factors and psychiatric outcomes and highlights the need for further research to elucidate the specific mechanisms underlying the interplay between *PER3* gene polymorphism and mental health outcomes. Karthikeyan R et al. reported variations in the frequency distribution of genotypes and alleles of the *PER3* gene in bipolar disorder (BD) among the South Indian population, which is similar to our study. Also, wake and sleep disturbances have been found among BD [10].

Golalipour et al. reported that individuals with the *PER3* 4-repeat allele and *PER3* 4/4 genotype showed a correlation with the occurrence of multiple sclerosis [19]. However, no significant variation was reported in the allele frequencies of the PER3 gene among obese and non-obese subjects [20]. Additionally, no variations were detected in patients with chronic heart disease compared to the control group regarding *PER3* genotype and allele frequencies. This suggests that *PER3* gene polymorphism may not be associated with chronic heart disease susceptibility or progression in the studied population. Further research is warranted to explore other genetic factors and potential mechanisms underlying the development and progression of chronic heart disease [21]. In a meta-analysis conducted by Geng et al., it was reported that individuals harboring the *PER3* five-repeated allele demonstrate a moderate increase in cancer risk compared to those carrying the four-repeated allele [22]. Similarly, Alexander et al. found in their study that individuals with the *PER3* five-repeated variant may display heightened susceptibility to the development of colorectal adenomas [15].

However, according to the findings of Yeg˘in et al., no association was observed between the *PER3* variant and bladder carcinoma [23]. This study is subject to several constraints that warrant consideration. Firstly, the investigation solely focused on one specific variation of the *PER3* gene, overlooking the potential contributions of other variations that may influence the progression of FMS. Given the genetic complexity of FMS, exploring a broader spectrum of *PER3* gene variations is essential to comprehensively elucidate its role in the disorder's pathogenesis. Additionally, the study did not delve into gene-gene and gene-environment interactions associated with the identified variant. Understanding how different genetic variants interact with each other and with environmental factors could provide valuable insights into the multifaceted nature of FMS and its etiology. Furthermore, the lack of assessment for the expression level of *PER3* represents another constraint of this study. Evaluating the expression level of *PER3* could offer valuable information regarding its functional significance in FMS development and progression. Integrating expression level analysis into future investigations may provide a more comprehensive understanding of the molecular mechanisms underlying FMS and the role of *PER3* gene variation therein.

The study has several notable strengths and limitations. A key strength is that it represents the first investigation exploring the association between *PER3* gene polymorphism and FMS, particularly in North Indian women, addressing a gap in genetic studies of circadian rhythm-related genes in this demographic. The robust sample selection, validated questionnaire, and strict quality control during PCR further enhance the study's reliability. Additionally, the focus on a relatively homogenous population minimizes confounding effects due to genetic variability. However, certain limitations warrant discussion. The study's focus on a single polymorphism in the *PER3* gene limits the scope of genetic insight, as other polymorphisms or genes involved in circadian regulation may also contribute to FMS pathogenesis. The lack of expression-level analysis of the *PER3* gene prevents understanding its functional impact on circadian rhythms in FMS. Furthermore, the sample size, while adequate for initial insights, may limit broader generalizability to other populations. Future research addressing these gaps is essential.

## Conclusions

Our study is the first to investigate the molecular genetics of *PER3* gene polymorphism in FMS, focusing on North Indian women. The findings reveal a significant association between *PER3* gene variations and FMS susceptibility, marking a crucial advancement in understanding the disorder's etiology within this population. The results suggest that* PER3* gene polymorphism, linked to circadian rhythm regulation, may influence FMS development by affecting sleep-wake patterns, hormonal balance, and pain perception. This highlights the *PER3* gene as a potential predictive marker for FMS pathogenesis and underscores the importance of genetic factors in disease assessment and management. Clinically, integrating* PER3* polymorphism into diagnostic frameworks could aid personalized treatment strategies, improving therapeutic outcomes. Beyond FMS, this study emphasizes the broader relevance of *CLOCK* genes in human health and disease, paving the way for future research on circadian rhythm disorders and novel therapeutic targets.

## Appendices

Appendix 1

General Assessment Questionnaire

1-Patients ID.: …………………………..       

2-Date of registration:……………………………….     

3-Place of residence: Urban=1, Rural=2……………………….   

4-Patient’s name: ………………………………….

5-Age:…………………..

6-Date of birth: (dd/mm/yy): ………………….  

7-Father’s name: …………………….

8-Religion: Hindu=1, Muslim=2, Sikh=3, Christian=4………………….   

9-Address:……………………….

10-Contact No. : ………………..

11-Email:…………………………

12-History of patient:

A- Have you lost your weight (Yes=1, No=2)………………..

B- Do you have frequent awakenings during the night? (Yes=1, No=2)……………………………………………

C- Family history of FMS: (Yes: 1, No: 2)……………………….

D- Sleep status:

Sound sleep: Yes=1, No=2………………..

Disturbed sleep: Yes=1, No=2…………………….

13- Present History of FMS:

A- Morning stiffness: Yes=1, No=2…………………..

B- Morning fatigue: Yes=1, No=2……………….

C- Headache: Yes=1, No=2…………………

D- Fever: Yes=1, No=2…………………

E- Abdominal pain: Yes=1, No=2……………..

F- Do you have any change in appetite? Yes=1, No=2………………..

G- Frequent awakening at night: Yes=1, No=2……………………

H- Lack of energy: Yes=1, No=2…………………………

I- Disequilibrium in: Yes=1, No=2……………………….

(Climbing stairs)………………………….

J- Jaw pain: Yes=1, No=2…………………………

K- Muscle twitching: Yes=1, No=2……………………………………

14- Clinical assessment:

Widespread pain history: (>3 months=1, >6 months=2, >1 year=3)………………….

Tender points examination: (11/18)

1)- Low cervical: Pain at the anterior aspect of intertransverse spaces at C5-C7. (Yes=1, No=2)………..

2)- Second Rib: Pain at costochondral junction just lateral to the junction on the upper surface. (Yes=1, No=2)…………

3)- Lateral epicondyle: Pain at points 2 cm distal to epichondyles. (Yes=1, No=2)………..

4)- Knee: Pain at medial fat pad proximal to joint line. (Yes=1, No=2)………….

5)- Occiput: Pain at suboccipital muscle insertion: (Yes=1, No=2)…………..

6)- Trapezius: Pain at mid-point of upper border. (Yes=1, No=2)……………..

7)- Supraspinatus: Pain above the scapula spine near the medial border. (Yes=1,No=2)…..

8)- Gluteal: Pain in upper outer quadrants of buttocks in anterior fold of muscle: (Yes=1, No=2)…………..

9)- Greater trochanter: Pain at posterior region of trochanteric prominence. (Yes=1, No=2)……

General assessment:

15)- During the first half hour after waking, how tired do you feel?            

(Very tired=1, fairly tired=2, fairly refreshed=3, very refreshed=4)……………..

16)- When you go to bed at night, at what level of tiredness are you?

(Not tired=1, a little tired=2, fairly tired=3, very tired=4)…………….

17)- Do you feel short of breath during sleep?

(Never=1, Sometime=2, Often=3)…………………

18)- Tobacco chewing (Yes=1, No=2)……………

29)-  Family history of subject’s 

20)- Have you taken any health supplements before? If yes, name them and for how long………………………………..

21)-If taken any medications, name them……………….

22)- Physical examination-

       a). Weight (in Kg)………………

       b).Height (in cm)………………

Annexure-2

Fibromyalgia Impact Questionnaire Revised (FIQR)

Domain 1: For each of the following nine questions, check the one box that best indicates how much your fibromyalgia made it difficult to do each of the following activities over the past 7 days:

Brush or comb your hair. No difficulty (1, 2, 3, 4, 5, 6, 7, 8, 9, 10) Very difficult

Walk continuously for 20 minutes. No difficulty (1, 2, 3, 4, 5, 6, 7, 8, 9, 10) Very difficult

Prepare a homemade meal. No difficulty (1, 2, 3, 4, 5, 6, 7, 8, 9, 10) Very difficult

Vacuum, scrub, or sweep floors. No difficulty (1, 2, 3, 4, 5, 6, 7, 8, 9, 10) Very difficult

Lift and carry a bag full of groceries. No difficulty (1, 2, 3, 4, 5, 6, 7, 8, 9, 10) Very difficult

Climb one flight of stairs. No difficulty (1, 2, 3, 4, 5, 6, 7, 8, 9, 10) Very difficult

Change bed sheets. No difficulty (1, 2, 3, 4, 5, 6, 7, 8, 9, 10) Very difficult

Sit in a chair for 45 minutes. No difficulty (1, 2, 3, 4, 5, 6, 7, 8, 9, 10) Very difficult

Go shopping for groceries. No difficulty (1, 2, 3, 4, 5, 6, 7, 8, 9, 10) Very difficult

Domain 2: For each of the following two questions, check the one box that best describes the overall impact of your fibromyalgia over the past seven days:

Fibromyalgia prevented me from accomplishing goals for the week. Never (1, 2, 3, 4, 5, 6, 7, 8, 9, 10) Always

I was completely overwhelmed by my fibromyalgia symptoms. Never (1, 2, 3, 4, 5, 6, 7, 8, 9, 10) Always

Domain 3: For each of the following 10 questions, check the one box that best indicates the intensity of your fibromyalgia symptoms over the past seven days:

Please rate your level of pain. No pain (1, 2, 3, 4, 5, 6, 7, 8, 9, 10) Unbearable pain

Please rate your level of energy. Lots of energy (1, 2, 3, 4, 5, 6, 7, 8, 9, 10) No energy

Please rate your level of stiffness. No stiffness (1, 2, 3, 4, 5, 6, 7, 8, 9, 10) Severe stiffness

Please rate the quality of your sleep. Awoke rested (1, 2, 3, 4, 5, 6, 7, 8, 9, 10) Awoke very tired

Please rate your level of depression. No depression (1, 2, 3, 4, 5, 6, 7, 8, 9, 10) Very depressed

Please rate your level of memory problems. Good memory (1, 2, 3, 4, 5, 6, 7, 8, 9, 10) Very poor memory

Please rate your level of anxiety. Not anxious (1, 2, 3, 4, 5, 6, 7, 8, 9, 10) Very anxious

Please rate your level of tenderness to touch. No tenderness (1, 2, 3, 4, 5, 6, 7, 8, 9, 10) Very tender

Please rate your level of balance problems. No imbalance (1, 2, 3, 4, 5, 6, 7, 8, 9, 10) Severe imbalance

Please rate your level of sensitivity to loud noises. No sensitivity (1, 2, 3, 4, 5, 6, 7, 8, 9, 10) Extreme sensitivity to bright lights, odors, and cold

Scoring: Step 1. Sum the scores for each of the three domains (function, overall, and symptoms). Step 2. Divide domain 1 score by three, divide domain 2 score by one (that is, it is unchanged), and divide domain score 3 by two. Step 3. Add the three resulting domain scores to obtain the total FIQR score.
